# Historical Atmospheric Lead Concentrations (1960‐1974) and Memory Problems Half a Century Later: Findings from Two Large, Independent Representative Samples

**DOI:** 10.1002/alz70860_101687

**Published:** 2025-12-23

**Authors:** Eric E Brown, Melissa Lombard, Alisha Chan, Joseph Ayotte, Scarlett Rakowska, Esme Fuller‐Thomson

**Affiliations:** ^1^ Centre for Addiction and Mental Health, Toronto, ON, Canada; ^2^ University of Toronto, Toronto, ON, Canada; ^3^ United States Geological Survey, Pembroke, NH, USA

## Abstract

**Background:**

Lead exposure has adverse impacts on cognition and dementia risk factors such as hypertension. A major historical source of lead exposure was via atmospheric pollution due to leaded gasoline.

**Method:**

We mapped historical atmospheric lead levels (HALL) in the contiguous US using measurements obtained by the US EPA from 1960 to 1974, a period of high leaded gasoline combustion. We extrapolated HALL by kriging and calculated mean HALL for each public use microdata area (PUMA). Our analyses include only PUMAs which contained at least one lead measurement in 1960‐74.

We obtained individual‐level data of self‐reported memory problems, from the American Community Survey (ACS) in two time periods, 2012‐2017 (*n* = 368,208) and 2018‐2021 (*n* = 276,476). The samples were restricted to respondents aged 65 and older living in their natal state.

We calculated odds ratios using HALL as the exposure variable and self‐reported memory problems as the outcome, controlling for respondents’ age, sex, race/ethnicity, and education. We used individuals in PUMAs with the lowest HALL (< 0.4 µg/m^3^) as reference in comparison to those in PUMAs with moderate (0.4‐.79 µg/m^3^), high (0.8‐1.19 µg/m^3^) and extremely high HALL (>=1.2 µg/m^3^).

**Result:**

In the 2012‐2017 ACS, in comparison to older adults living in PUMAs with the lowest HALL, the odds of reported memory impairment were higher in those in PUMAs with moderate (Odds ratio (OR)=1.21; 95% CI=1.17‐1.25), high (OR=1.21; 95% CI=1.17‐1.25) and extremely high HALL levels (OR=1.19; 95% CI=1.13‐1.25). We replicated the study using 2018‐2021 ACS data and found comparable outcomes for older adults living in PUMAs with moderate (OR = 1.17; 95% CI=1.12‐1.21), high (OR=1.20; 95% CI=1.16‐1.25) and very high HALL (OR=1.22; 95% CI=1.15‐1.29).

**Conclusion:**

We observed in two very large (*n* >250,000) independent representative samples that older adults had approximately 20% higher odds of reporting memory problems if they lived in PUMAs that had HALL > 0.4 µg/m^3^compared to <0.4 µg/m^3^. This adds to the evidence implicating lasting health outcomes, including cognition, due to earlier life lead exposure from air pollution. The precipitous decline in atmospheric lead exposure in the last quarter of the 20^th^ century may help to explain the declining incidence of dementia in the US.

